# Spinal pseudoarthrosis following osteoporotic vertebral fracture: prevalence, risk factors, and influence on patients’ activities of daily living 1 year after injury

**DOI:** 10.1007/s11657-023-01236-8

**Published:** 2023-03-29

**Authors:** Norimitsu Wakao, Yoshihito Sakai, Tsuyoshi Watanabe, Naoaki Osada, Takaya Sugiura, Hiroki Iida, Yuto Ozawa, Kenta Murotani

**Affiliations:** 1 Department of Orthopedic Surgery National Center for Geriatrics and Gerontoloty, Obu, Aichi Japan; 2 Department of Orthopedic Surgery, Nagoya University Graduate School of Medicine, Nagoya, Aichi Japan; 3 Biostatistics Center, Kurume University, Kurume, Fukuoka Japan

**Keywords:** Osteoporotic vertebral fracture, Pseudoarthrosis, Prevalence, Risk factor, ADL, Gait ability, Multivariate analysis, MRI, BKP, Conservative therapy

## Abstract

**Purpose:**

To investigate the prevalence and risk factors and influence of pseudoarthrosis on activities of daily living (ADL) of patients with osteoporotic vertebral fracture (OVF).

**Methods:**

Spinal pseudoarthrosis is defined as the presence of a cleft in the vertebral body on a lateral X-ray image in the sitting position at 1 year after admission. Of the total 684 patients treated for OVF between January 2012 and February 2019 at our institution, 551 patients (mean age, 81.9 years; a male-to-female ratio, 152:399) who could be followed up to 1 year were included in this study. Prevalence, risk factors, and influence of pseudoarthrosis on the ADL of patients as well as fracture type and location were investigated. Pseudoarthrosis was set as the objective variable. Total bone mineral density, skeletal muscle mass index, sex, age, history of osteoporosis treatment, presence of dementia, vertebral kyphosis angle, fracture type (presence of posterior wall injury), degree of independence before admission, history of steroid use, albumin level, renal function, presence of diabetes, and diffuse idiopathic skeletal hyperostosis were set as explanatory variables for multivariate analysis of the influence of pseudoarthrosis on the walking ability and ADL independence before and 1 year after OVF.

**Results:**

In total, 54 (9.8%) patients were diagnosed with pseudarthrosis 1 year after injury (mean age, 81.3 ± 6.5 years; male-to-female ratio, 18:36). BKP was performed in nine patients who did not develop pseudoarthrosis after 1 year. In the multivariate analysis, only the presence of posterior wall injury was significantly correlated with the presence of pseudoarthrosis (OR = 2.059, *p* = 0.039). No significant difference was found between the pseudarthrosis group and the non-pseudarthrosis group in terms of walking ability and ADL independence at 1 year.

**Conclusions:**

The prevalence of pseudoarthrosis following OVF was 9.8%, and its risk factor was posterior wall injury. The BKP group was not included in the pseudoarthrosis group, which may have led to an underestimation of the prevalence of pseudoarthrosis.

**Summary:**

The prevalence, risk factors, and influence of spinal pseudoarthrosis on patients’ ADL following osteoporotic vertebral fracture (OVF) were investigated. Pseudoarthrosis occurs in 9.8% 1 year after the injury in patients with OVF. Posterior wall injury was the risk factor of pseudoarthrosis.

## Introduction

**Fig. 1:**
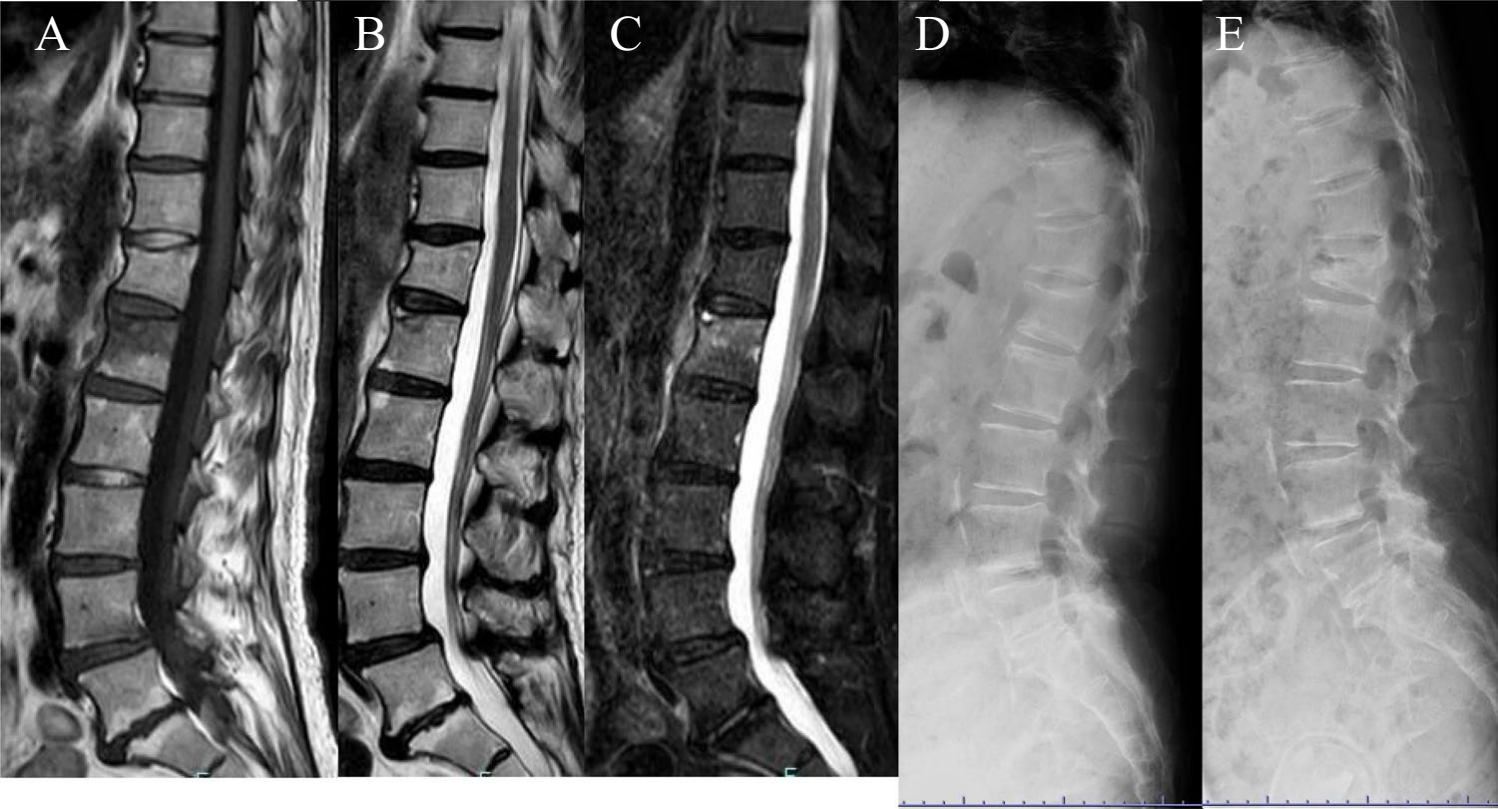
Images of patients with pseudoarthrosis of L1 fracture. MR T1-weighted image (**A**), T2-weighted image (**B**), and STIR image (**C**) at injury. **D** shows lateral radiogram at injury. E shows lateral radiogram 1 year after the injury

The prevalence of osteoporotic vertebral fractures (OVFs) is increasing with the increase in elderly population [1–4]. Bone fusion and stabilization are achieved naturally in most OVF cases [5, 6]. However, orthopedic doctors occasionally come across patients in whom early stabilization has not been achieved [7–13], and the technical terms for this pathology have not been defined clearly. The Japanese clinical guideline for OVFs was revised in 2012, and pseudoarthrosis of the spine was defined as the lack of visible signs of healing 12 months after the onset of the fracture (Fig. 1). In long-term follow-up of patients with OVFs, pseudoarthrosis developed in many cases 1 year after injury without any adverse complications [10, 14], and the clinical significance and frequency of spinal pseudoarthrosis remain unclear. This study aimed to clarify the clinical significance of vertebral pseudoarthrosis by investigating the rate of spinal pseudoarthrosis following OVFs, risk factors for pseudoarthrosis, and influence of pseudoarthrosis on walking ability and independence in daily living after 1 year in patients treated at our institution.

## Materials and methods

**Fig. 2:**
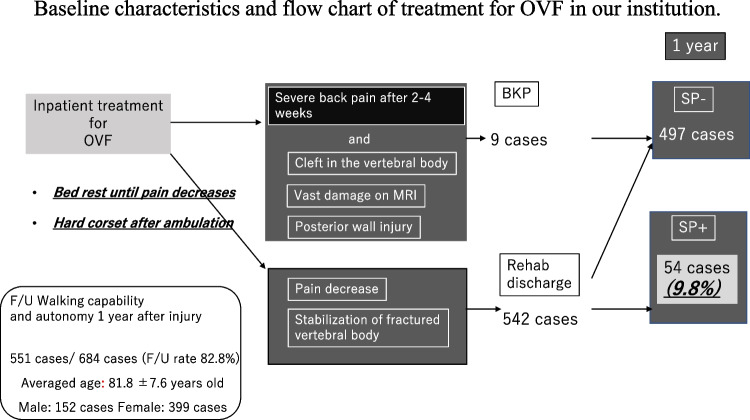
Baseline characteristics and flowchart of treatment for OVF

Eligible subjects were patients with acute OVFs who were hospitalized and treated in our institution between January 2012 and February 2019. In our institution, patients with acute OVFs are treated as inpatients (Fig. 2). After hospitalization, the treatment strategy for OVFs is as follows: (1) bed rest and rehabilitation on the bed until patients wear a made-to-order brace, (2) bed rest until the pain during body movements is relieved (whether or not the patient can change positions by himself/herself), (3) wearing a brace of sufficient length and starting walking training if the pain during body movement is improved, (4) starting or continuing osteoporosis treatment, (5) balloon kyphoplasty (BKP) should be considered if pain with movements does not improve after 2–4 weeks, and vertebral body damage is considered severe on imaging (T1, diffuse low on magnetic resonance imaging (MRI); T2, fluid accumulation, posterior wall injury on MRI) [15].

The survey items were as follows: (1) presence of pseudoarthrosis (Fig. 1) (spinal pseudoarthrosis is defined as the presence of a cleft in the vertebral body on a lateral X-ray image in the sitting position at 1 year after admission); (2) ADL assessment included walking ability (independent/assisted/unable to walk) before and 1 year after injury and level of ADL independence (independent/non-independent: The level of independence in daily living was determined based on the classification of care level evaluated by long-term care insurance system in Japan, which all seniors 65 years of age and older are enrolled in.); and (3) patient background factors such as whole-body bone mass measured by dual-energy X-ray absorptiometry, skeletal muscle mass index, sex, age, history of osteoporosis treatment before admission, presence of dementia (The presence or absence of dementia was determined by the mini-mental state examination (MMSE); a score of 27 or higher was considered normal, and a score of less than 27 was diagnosed as dementia.), kyphotic angle of the fractured vertebrae, presence of posterior wall injury on MRI, degree of independence before admission, history of steroid medication, albumin level, renal function (eGFR), diabetes, and diffuse idiopathic skeletal hyperostosis (DISH). The following data were also obtained: degree of independence, steroid history, albumin level, eGFR, presence of diabetes treatment, and presence of DISH (fusion of consecutive four vertebrae on frontal and lateral X-ray images of the vertebrae).

### Statistical analysis

From these survey items, we first investigated the prevalence of pseudoarthrosis. Then, the influence of pseudoarthrosis on walking ability was investigated by comparing the pseudoarthrosis group and the non-pseudoarthrosis group (Mann–Whitney U test). Factors influencing pseudoarthrosis were investigated by univariate and multivariate logistic analyses with pseudoarthrosis as the objective variable and the above survey items as explanatory variables. Items with *p* < 0.10 in the univariate logistic analysis were selected, and multivariate logistic analysis was performed using these variables as explanatory variables. SAS 9.4 (SAS Institute Inc., Cary, NC, USA) was used, and the significance level was set at *p* = 0.05.

## Results

**Fig. 3:**
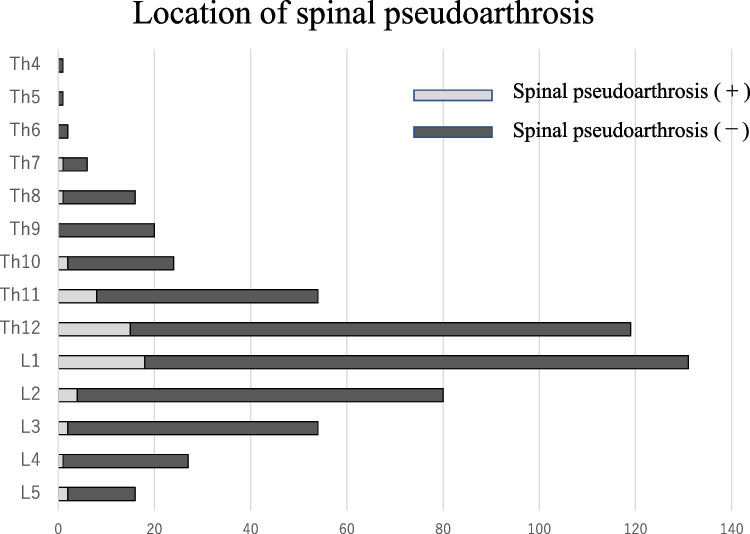
Fracture location and pseudoarthrosis

**Fig. 4:**
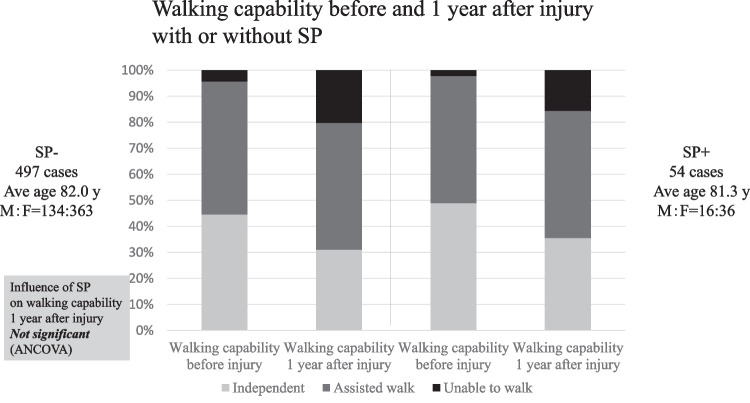
Results of walking capability before and 1 year after injury in the pseudoarthrosis group and non-pseudoarthrosis group

**Fig. 5:**
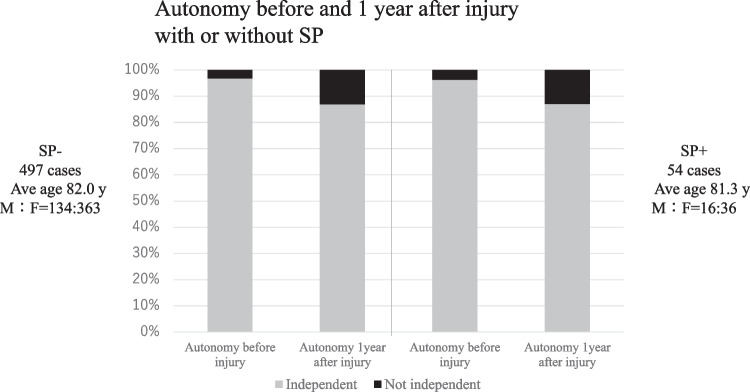
Results of autonomy before and 1 year after injury in the pseudoarthrosis group and non-pseudoarthrosis group

**Fig. 6:**
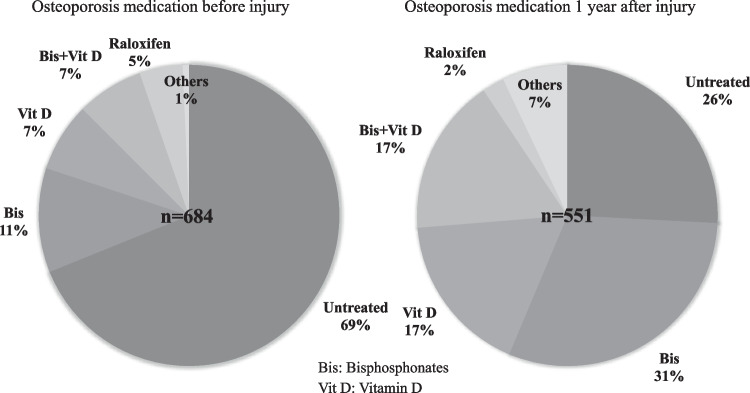
Osteoporosis medication before and 1 year after injury

Of the 684 patients treated for OVF between January 2012 and February 2019, 551 patients (mean age, 81.8 ± 7.6 years; male-to-female ratio, 152:399) were followed up 1 year after the injury and eligible in this study. BKP was performed in nine patients who had poor pain relief after conservative treatment and hospitalization and developed advanced vertebral damage on imaging. These patients did not develop pseudoarthrosis after 1 year. The mean duration of bed rest after admission for the 542 patients who did not undergo BKP was 6.3 days. No patients developed osteoporotic-delayed vertebral collapse (ODVC) and paralysis requiring surgical treatment by 1 year. Among all patients, 54 (9.8%) were diagnosed with pseudoarthrosis after 1 year (mean age, 81.3 ± 6.5 years; male-to-female ratio, 18:36) (Table 1). Fracture location with and without pseudoarthrosis is shown in Fig. 3. Fractures occurred more frequently in the thoracolumbar site and less frequently in the upper thoracic and lower lumbar spine. The number of pseudoarthrosis was also higher in the thoracolumbar site but could not be statistically significant due to differences of number of occurrences. The walking ability of the patients before and 1 year after injury in the pseudoarthrosis and non-pseudoarthrosis groups is shown in Fig. 4. Although a certain number of patients in each group had decreased walking ability and became unable to walk, the presence of a pseudoarthrosis did not significantly affect the decrease in walking ability (*p* = 0.52). Similarly, the results for ADL independence are shown in Fig. 5. Although a certain number of patients in each group showed a decrease in ADL independence, as with the walking ability, the presence of a pseudoarthrosis had no significant effect on the decrease in ADL independence (*p* = 0.48). The results of the univariate and multivariate analyses of factors influencing pseudoarthrosis are shown in Table 2. In the univariate analysis, the vertebral kyphosis angle in patients with fracture at admission (OR = 0.968, *p* = 0.009) and posterior wall injury (OR = 2.561, *p* = 0.005) were significant (OR = 2.059, *p* = 0.039); however, only posterior wall injury was significantly correlated with the presence of pseudoarthrosis in the multivariate analysis.　Figure 6 shows the administration of osteoporosis medication before injury and 1 year after injury.

**Table 1:**
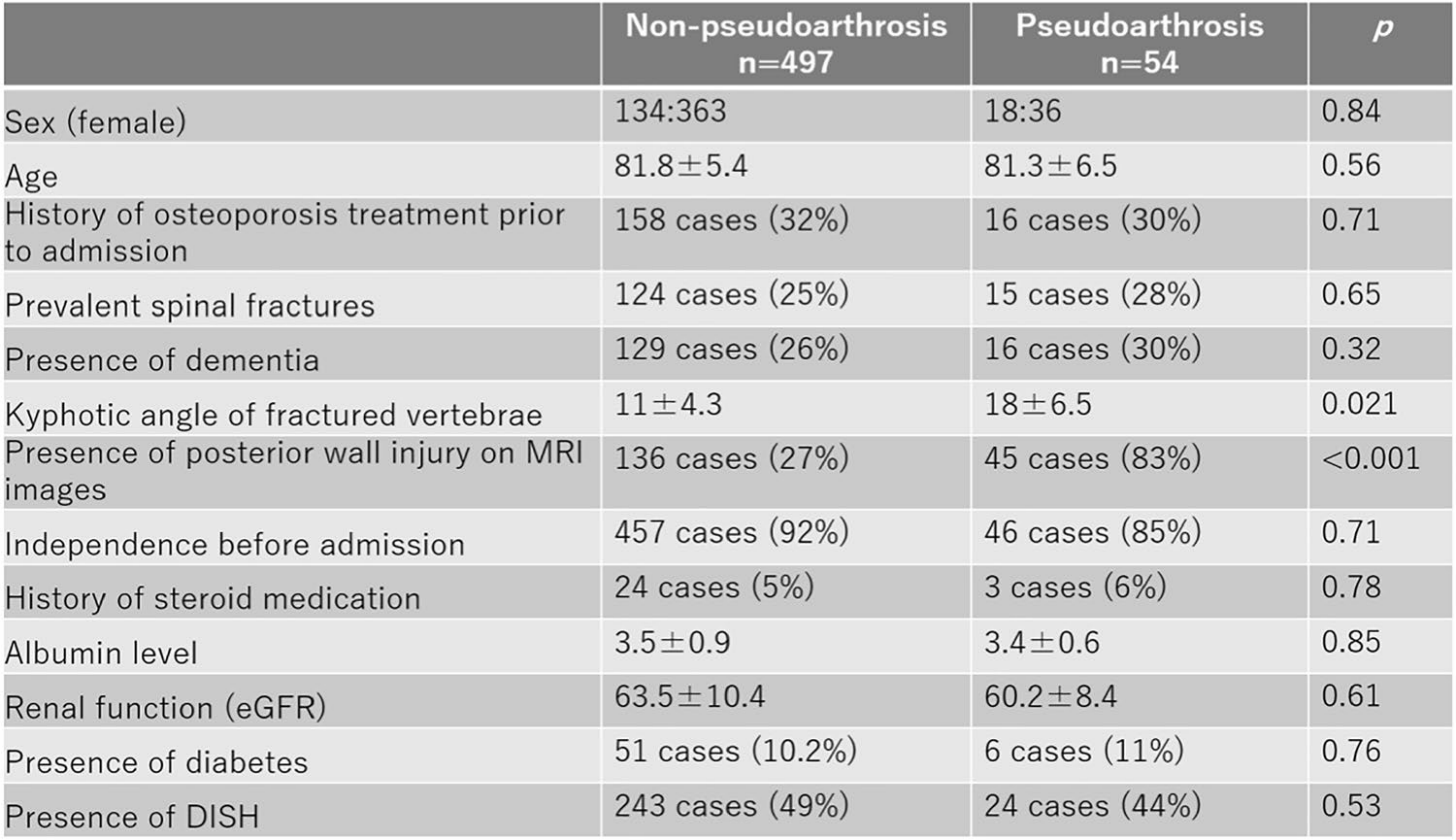
Comparison of pseudoarthrosis and non-pseudoarthrosis groups

**Table 2:**
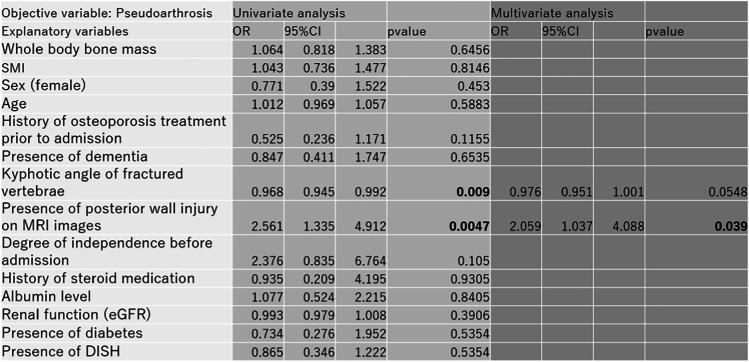
Results of univariate and multivariate analyses

Abbreviation: SMI; Skeletal muscle mass index eGFR; estimated glomerular filtration rate,DISH; diffuse idiopathic skeletal hyperostosis

## Discussion

Based on the results of this study, the pseudoarthrosis rate was 9.8%. Compared with previous reports in Japan, the frequency was slightly low [7, 8, 10, 14, 16]. This result was possibly influenced by two factors. First, our hospitalization policy for all patients with OVFs has led to good outcomes of conservative therapy. Inpatient treatment for OVFs is available at a few facilities and is especially difficult in high care units in emergency hospitals. This may naturally lead to missed cases, delayed diagnosis, inadequate initial treatment, and inappropriate orthotic prescriptions. Second, BKP was performed in nine cases [15]. These nine patients had vertebral stabilization after 1 year and did not belong to the pseudoarthrosis group. Even considering these differences in study backgrounds, it is confirmed that pseudoarthrosis occurs 1 year after OVF in approximately 10–20% of cases.

Non-union, fusion failure, and pseudoarthrosis following OVFs are considered factors with poor functional prognosis; indeed, some clinical studies have investigated patients with residual clefts in the vertebral body after a certain period of conservative treatment [7, 10, 12, 13, 16, 17]. However, in long-term follow-up of fracture cases, there are patients who developed pseudoarthrosis after 1 year without any adverse complications. When spine surgeons reviewed cases of ODVC requiring major surgical treatment [9], all cases involved vertebral instability several months after the injury, and vertebral instability does not necessarily result in adverse events such as intractable pain and paralysis. ODVC occurs within 6 months after the injury, and non-fused vertebrae at 1 year after injury (pseudoarthrosis) may not result in significant adverse events. Based on these clinical questions and characteristics, this study focused on two clinical influences of pseudoarthrosis: walking ability and independence in daily living after 1 year. The results showed that pseudoarthrosis did not have a significant negative effect on both walking ability and independence in daily living. This is new knowledge that has not been reported previously. Although previous studies have mentioned back pain and health-related quality of life and have reported inferior outcomes in the pseudoarthrosis group compared with the non-pseudoarthrosis group, influences on walking ability or independence in daily living were not investigated. The results of this study are of great clinical significance from the viewpoint that the actual functional prognosis after 1 year is more important in the case group of patients aged 80 years, since diseases other than OVFs have a diverse effect.

The results of the multivariate analysis showed that only posterior wall injury significantly correlated with the presence of pseudoarthrosis, which was also consistent with previous reports [7, 8, 10, 13, 14]. Recently, MR images in the early phase of OVFs predict pseudoarthrosis [14, 15], and diffuse low on T1-weighted images and diffuse low with fluid retention on T2-weighted images may be useful prognostic factors for poorer clinical outcome following OVFs. Our study focused on examining more confounding factors and examined in detail patient factors that have not been examined in many previous case studies. The result that only the posterior vertebral wall injury on MRI was significant is very interesting and reinforces the possibility that the severity of the fracture and the limitation of conservative treatment are determined at the time of diagnosis, as has been reported in the past. The authors plan to add MRI signal changes as one of the explanatory variables in the future. Patients with OVF and posterior wall injuries are predisposed to have difficulty obtaining subsequent vertebral fusion, and, if OVF-derived symptoms do not improve after careful conservative treatment, minimally invasive surgical intervention, including BKP, is necessary before severe vertebral deformity or neurological manifest develops [15].

## Strength and limitation of this study

The study included many OVF cases, and 551 of 684 (80.6%) were functionally evaluated 1 year after injury. The number of cases and the small number of dropouts are strengths of the study, which increase the reliability of the analysis results but also have limitations. First, the BKP group, which is supposed to have a poor functional prognosis, was not included in the pseudoarthrosis group. This may have led to an underestimation of the prevalence of pseudoarthrosis in this study. Second, assessment of the effect of pseudoarthrosis on the patient was limited. Essentially, there were not many cases in which OVF caused significant functional decline 1 year after the injury. Specifically, cases in which functional assessment at 1 year was not possible (133 cases, 19.4% in this study) probably included deaths or cases in which patients had to be transferred or institutionalized to nursing homes due to functional decline. Furthermore, proof of functional decline due to OVF must exclude the influence of other diseases as much as possible. In the present study, the patients were evaluated in terms of walking ability and independence in daily living, but more detailed evaluation indices (pain, walking speed, walking distance, etc.) would have made a difference. Third, the method of evaluating pseudoarthrosis was limited. There is no uniform method for cleft evaluation 1 year after injury, and the pseudoarthrosis rate would be higher if computed tomography or functional radiographic imaging with anterior and posterior bending were used as evaluation methods. This study employed lateral radiography in the sitting position and a method of imaging that is thought to have the lowest pseudoarthrosis diagnosis rate, which may have missed true pseudoarthrosis.

## Conclusions

Spinal pseudoarthrosis, which occurs at a certain rate after OVFs, was investigated based on the revised clinical guideline for OVFs in 2012. Pseudoarthrosis was observed in 9.8% of all cases. The influence of pseudoarthrosis was examined in terms of two aspects: walking ability and independence in daily living, but no difference was found between the pseudoarthrosis group and the non-pseudoarthrosis group. The inclusion of nine patients who underwent BKP in the non-pseudarthrosis group might have led to bias. The only significant risk factor that affected pseudoarthrosis was posterior wall injury following OVF.

## Data Availability

Our data investigated in this study is available.
